# A Comprehensive Computer Aided Vaccine Design Approach to Propose a Multi-Epitopes Subunit Vaccine against Genus *Klebsiella* Using Pan-Genomics, Reverse Vaccinology, and Biophysical Techniques

**DOI:** 10.3390/vaccines9101087

**Published:** 2021-09-27

**Authors:** Khaled S. Allemailem

**Affiliations:** Department of Medical Laboratories, College of Applied Medical Sciences, Qassim University, Buraydah 51452, Saudi Arabia; k.allemailem@qu.edu.sa; Tel.: +966-536-333-777

**Keywords:** *Klebsiella*, pan-proteome, multi-epitopes vaccine, immunoinformatics, molecular dynamics simulations

## Abstract

*Klebsiella* is a genus of nosocomial bacterial pathogens and is placed in the most critical list of World Health Organization (WHO) for development of novel therapeutics. The pathogens of the genus are associated with high mortality and morbidity. Owing to their strong resistance profile against different classes of antibiotics and nonavailability of a licensed vaccine, urgent efforts are required to develop a novel vaccine candidate that can tackle all pathogenic species of the *Klebsiella* genus. The present study aims to design a broad-spectrum vaccine against all species of the *Klebsiella* genus with objectives to identify the core proteome of pathogen species, prioritize potential core vaccine proteins, analyze immunoinformatics of the vaccine proteins, construct a multi-epitopes vaccine, and provide its biophysical analysis. Herein, we investigated all reference species of the genus to reveal their core proteome. The core proteins were then subjected to multiple reverse vaccinology checks that are mandatory for the prioritization of potential vaccine candidates. Two proteins (TonB-dependent siderophore receptor and siderophore enterobactin receptor FepA) were found to fulfill all vaccine parameters. Both these proteins harbor several potent B-cell-derived T-cell epitopes that are antigenic, nonallergic, nontoxic, virulent, water soluble, IFN-γ producer, and efficient binder of DRB*0101 allele. The selected epitopes were modeled into a multi-epitope peptide comprising linkers and Cholera Toxin B adjuvant. For docking with innate immune and MHC receptors and afterward molecular dynamics simulations and binding free energy analysis, the vaccine structure was modeled for tertiary structure and refined for structural errors. To assess the binding affinity and presentation of the designed vaccine construct, binding mode and interactions analysis were performed using molecular docking and molecular dynamics simulation techniques. These biophysical approaches illustrated the vaccine as a good binder to the immune receptors and revealed robust interactions energies. The vaccine sequence was further translated to nucleotide sequence and cloned into an appropriate vector for expressing it at high rate in *Escherichia coli* K12 strain. In addition, the vaccine was illustrated to generate a good level of primary, secondary, and tertiary immune responses, proving good immunogenicity of the vaccine. Based on the reported results, the vaccine can be a good candidate to be evaluated for effectiveness in wet laboratory validation studies.

## 1. Introduction

The genus *Klebsiella* is ubiquitous in nature and comprises Gram-negative, rod shaped, and oxidase negative bacteria, and has a prominent polysaccharide capsule [1]. The genus is named after Edwin Klebs, a German–Swiss microbiologist. Species of this genus are part of animal and humans normal flora found in the mouth, nose, and intestines [2]. They are 0.5 to 5.0 µm long and 0.3 to 1.5 µm wide [3]. From a medical perspective, the genus contains the following important species: *K. aerogenes, K. granulomatis, K. oxytoca, K. michiganensis, K. pneumoniae* (*K. p.* subsp. *Ozaenae, K. p.* subsp. *Ozaenae*, *K. p.* subsp. *Pneumoniae, K. p.* subsp. *Rhinoscleromatis), K. quasipneumoniae (K. q.* subsp. *quasipneumoniae, K. q.* subsp. *similipneumoniae), K. grimontii, K. variicola,* and *K. planticola* [4]. *Klebsiella* species are responsible for different diseases; notably, they cause pneumonia but also urinary tract infections, meningitis, diarrhea, sepsis, and peritonitis and soft tissue infections [5]. The bacterial species mentioned are also implicated in spondyloarthropathies and ankylosing spondylitis pathogenesis and in most cases, *K. pneumoniae* and *K. oxytoca* are the main causative agents of *Klebsiella*-associated diseases [6]. *Klebsiella pneumoniae* is of prime concern as it is a major cause of nosocomial infections and the isolated clinical strains are found to be resistant against a broad range of antibiotics [7]. The pathogen is also responsible for community acquired infection especially in individuals with underlying conditions or who are immunocompromised [8].

β-lactam antibiotics are often used to treat *K. pneumoniae* infections and resistance emergence against β-lactam made the treatment options limited [9]. Moreover, the *K. pneumoniae* is naturally more resistant as they produce more β-lactamases including extended-spectrum β-lactamases (ESBLs) [10]. These enzymes are capable of hydrolyzing cephalosporin and penicillin thus making them ineffective. Consequently, the meropenem and imipenem (carbapenem antibiotics) are applied for ESBL-producing *Klebsiella* species [11]. However, the heavy use of antibiotics in clinical settings pushed the gut commensal *Klebsiella* species to acquire carbapenem-resistance [12]. These strains harbor carbapenemase, a plasmid encoded enzyme, to hydrolyze all carbapenems and make the strains super resistant to all β-lactam antibiotics [13]. The two most prevalent carbapenems *enzymes* include New Delhi metallo-beta-lactamase (NDM-1) and *K. pneumoniae* carbapenemase (KPC) [14]. Carbapenem-resistant *Klebsiella* species are the common carbapenem-resistant Enterobacteriaceae and are mainly responsible for high morbidity and mortality [15]. The epidemiology success of KPC–*K. pneumoniae* strains is evident by the strain ST258 that is abundant worldwide and account for ∼70% carbapenem resistance in the United States [16]. This multi-drug resistance by *K. pneumoniae* is a major hurdle in treating carbapenem-resistant *K. pneumoniae* isolates in fatal cases where the bacteria were resistant to 26 antibiotics [17]. The last line of therapy including tigecycline and colistin when used in combination therapy has been revealed successful [18]. Nevertheless, because of nephrotoxicity caused by colistin, such treatment is not a good option [19]. In addition, the use of excessive colistin can result in the emergence of colistin resistance.

During the last several years, many trials have been conducted to evaluate *K. pneumoniae* vaccines; however, no licensed vaccine is currently available [20]. Thus, urgent efforts are required to combat this public health crisis. A 24 valent capsular polysaccharide (SPC) vaccine in trials was unveiled to provide 80% of protection against *K. pneumoniae* isolates, but the purified polysaccharide vaccines are weak immunogens [20]. Vaccines containing four common *Klebsiella* O polysaccharides have been proposed as conjugate vaccines that reported good immunogenicity in mice studies [20]. Traditional immunization studies involve isolation of pathogen specific antigen, and reinjection into subject organisms to evaluate protective immune responses [21]. Such procedures are costly and time consuming. One disadvantage of the conventional vaccinology is that it is not appropriate for pathogens such as *Klebsiella* species, as the genus comprises diverse species each having variety of strains [22]. The search for finding conserved antigens, together with their processing and assessment, would be exhaustive. Significant advancements in DNA sequencing technologies, along with system biology, genomics, and proteomics have allowed scientists to gain a better understanding of vaccine design [23]. By using this integrated approach, it is now possible to test all antigens of a pathogen for their immunogenicity. Computational based genome mining to identify potential vaccine proteins is now done through a technique called reverse vaccinology [24]. Meningococcus B was the first pathogen that successfully was addressed by reverse vaccinology. The bacterium surface proteins are extremely variable and homology exists between the pathogen capsular polysaccharide and human self-antigens [21]. Genomics based methods predicted 90 previously unknown antigens. Meningococcus B and 29 out of them were found to stimulate antibodies that were able to kill the bacteria. In addition, the reverse vaccinology technique is used for development of protein-based vaccines against *Streptococcus pneumoniae* and *Staphylococcus* [21]. This technique can be integrated with several other approaches such as pang-genomics, immunoinformatics, and different biophysical analysis to construct a novel multi-epitope peptide [25,26]. The core vaccine proteins were identified using pan-proteome analysis and used for broad spectrum epitopes prioritization [23]. Further, it was ensured that the selected epitopes are vital for the pathogen survival that are not human homologs, and are exposed to the host immune system for efficient recognition and processing. Ideally, conserved epitopes that were found to generate specific B- and T-cell responses were selected to enable indication of pathogen specific immune responses.

## 2. Methodology

**Figure 1:**
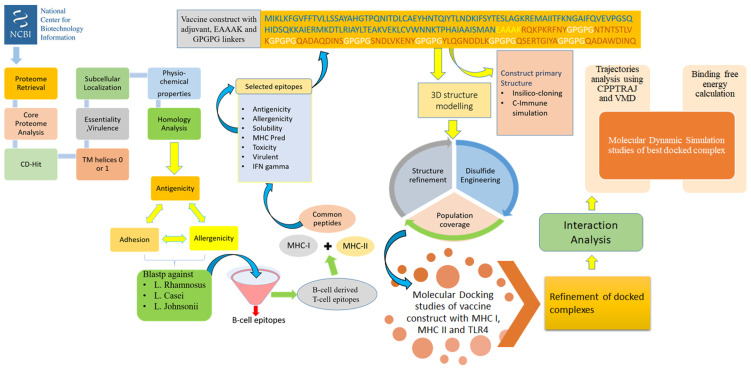
Complete flow of work done to prioritize potential vaccine candidates from pan-proteome of *Klebsiella* species.

The methodology of the study can be explained in a stepwise fashion and is illustrated in Figure 1.

### 2.1. Proteome Retrieval and Subcellular Localization

Primarily, the reference proteomic data of all the seven species of *Klebsiella* were retrieved from the NCBI database [27]. These seven species of *Klebsiella* include *K. aerogenes*, *K. oxytoca*, *K. michiganansis*, *K. pnemoniae*, *K. quasipneumoniae*, *K. grimontii,* and *K. variicola*. The proteomes were subjected to pan-proteome analysis to retrieve the core sequence. The Bacterial Pan Genome Analysis (BPGA) tool [28] was used to perform pan-proteome analysis. The core sequence retrieved through BPGA was then clustered through CD-Hit [29] to remove redundant proteins from the core sequence. The threshold was set at 0.5, so all the sequences having 50% similarity were clustered together. The subcellular localization of selected proteins was predicted through PSORTb 3.0 subcellular localization prediction tool [30]. The surface proteins were then filtered using several parameters to prioritize potential vaccine candidates for the pathogen [31,32,33,34].

### 2.2. Vaccine Candidate Prioritization Phase

Virulent proteins are a primary target for vaccine candidates because they play a significant role in pathogenesis [35,36]. The nonredundant set of proteins were used in BLASTp search against the core virulent factor database (VFDB) [37] and those having sequence identity ≥30% and bit score >100 were selected. Subsequently, the physiochemical properties of proteins were checked using ProtParam tool [38]. The proteins having molecular weight <110 kDa and thermostability index >40 are considered as ideal vaccine candidates as they can be easily purified [39,40]. The proteins were then subjected to transmembrane helices prediction by using HMMTOP 2.0 [41]. The proteins predicted to show (1 or 0) outside transmembrane helices were selected [42]. The shortlisted proteins were then BLAST against reference human proteome, which has “taxonomic ID: 9606” in the BLASTp of NCBI and those with E-value < 1, bit score >100 and sequence identity >30% were discarded [43]. All the homologous proteins were discarded to avoid the host’s autoimmune reactions. Antigenicity of the proteins was predicted next through VaxiJen 2.0 with the threshold of 0.4 [44]. All proteins depicting an antigenicity score >0.5 were selected as antigenic proteins and the other proteins were discarded. Thus, these proteins that are highly antigenic towards T-cell receptors and antibodies are potential vaccine targets. The next step was to predict the adhesive nature of selected protein candidates as proteins having adhesive property are effective meditators of prompting pathogenesis. The adhesive nature of proteins was checked using SPAAN server keeping all the settings at default [45]. The AllerTOP2.0 [46] was then used to check the allergenicity of proteins that had IC_50_ values ≤ 100 nM [43]. All the allergen proteins were removed to avoid allergic reactions, leaving only nonallergen proteins. Another BLASTp search of NCBI against *Lactobacillus rhamnosus*, *Lactobacillus casei*, and *Lactobacillus johnsonii* was performed to check homology of proteins with the mentioned probiotic species [32]. All the proteins that were showing no similarity were selected for epitope mapping analysis.

### 2.3. B-Cell and T-Cell Epitopes Prediction

Successively, the liner B-cells epitopes were then predicted by using Bepipred Linear Epitope Prediction 2.0 [47], which is accessible at Immune Epitope Database (IEDB) with a threshold of 0.5 [48]. The resulting peptides were then used in T-cell epitopes mapping in IEDB T-cell epitopes prediction tools to predict subsequences that bind to alleles of major histocompatibility complex (MHC) class I and II alleles [49]. The peptides with a percentile score <10 are high affinity binders [40]. The MHCPred 2.0 analysis [50] was then executed to calculate the binding affinity of the predicted epitopes for DRB*0101 allele and only those with IC_50_ values ≤ 100 nM were selected [43]. Antigenicity, allergenicity, and virulence of the common peptides finally selected were checked using VaxiJen [44], AllerTOP 2.0 [51], and VirulentPred [52], respectively. The solubility of peptides was then checked using the Innovagen tool, which is used as a peptide solubility calculator. All the peptides showing good water solubility were selected. Subsequently, ToxinPred [53] was used to check the toxicity of antigenic nonallergen virulent epitopes [54]. All the nontoxic peptides were selected and subjected to check whether these epitopes can prompt IFN-gamma or not using the IFN epitope server [55].

### 2.4. Designing and Processing of Vaccine Construct

The conventional vaccines using whole organism or large proteins engender nonspecific immune responses as it carries unnecessary antigenic load [56]. The development of a peptide vaccine, as a substitute to conventional vaccines, generates highly specific immune responses as it depends on short peptides that are free from allergenic reactions [34,57]. However, peptide vaccines are weakly immunogenic [58]. To overcome this problem, all the epitopes selected were fused using GPGPG linkers to induce an immune response sufficiently strong to combat pathogens. This multi-epitope vaccine construct was then joined to an adjuvant cholera toxin B (CTB) [59] using EAAAK linker. The computation of various chemical and physical parameters of the vaccine construct was calculated using the ExPASy Protparam tool [38]. It permits calculation of various parameters such as instability index, amino acid composition, theoretical pI, molecular weight, and grand average of hydropathicity (GRAVY)**.** After this, a file was created in which all the epitopes were added with alleles in order to check worldwide population coverage by additionally using population coverage, which is accessible at Immune Epitope Database (IEDB) [60]. The designed multi-epitopes vaccine construct should show extensive human population coverage. The multi-epitopes vaccine construct was subjected to tertiary structure predictions using 3Dpro of SCRATCH protein predictor [61]. The structure refinement was done through GalaxyRefine of GalaxyWeb [62,63].

### 2.5. Blind Docking Analysis

Blind molecular docking is executed to analyze and observe the binding affinity of multi-epitopes vaccine construct with immune receptors. If the designed vaccine construct is showing interactions with host receptors and immune cells, then good immune responses can be established. The blind docking of multi-epitopes vaccine construct was performed by choosing TLR4, MHC-I, and MHC-II as receptors in PatchDock server [64]. The 3D structure of TLR4, MHC-I, and MHC-II were retrieved from Protein Data Bank (PDB) using code 4G8A, 1I1Y, and 1KG0, respectively. Additionally, the PatchDock complexes were assembled through RMSD, which was set to default at 4.0 Å. The protein–protein docking output generated through PatchDock was further subjected for refinement by using a FireDock (Fast Interaction Refinement in Molecular Docking) [65]. The FireDock helps to provide an effective platform for producing refined PatchDock complexes. After this, all of the complexes that were showing lowest global energy were ranked at the top and subjected to further evaluation. UCSF Chimera 1.13.1 [66] was used to investigate intermolecular interactions among the immune receptors (TLR4, MHC-I, and MHC-II) and the vaccine construct.

### 2.6. Molecular Dynamics Simulation Assay

The molecular dynamics simulation assay helps to analyze the stability and dynamics of all the docked complexes in three steps: (a) system preparation, (b) preprocessing, and (c) production phase. A 100 ns simulation run for the docked complexes was performed using AMBER20 [67]. The first phase of the simulation was to prepare the complexes parameters using antechamber module. To solvate the complexes into TIP3P solvation box, the leap module of AMBER was used and the input value for size was set at 12 Å. To study the intermolecular and intramolecular interactions in molecular dynamics assay, a force field of ff14SB was applied [68]. Na^+^ ions as counter ions were incorporated into the system for neutralization of charges. In the second round of the preprocessing phase, energies of the systems were minimized: energy minimization of hydrogen atoms (500 cycles), water box (1000 cycles), complete system atoms (1000 cycles), and nonheavy atoms (300 cycles). Subsequently, using NVB ensemble, the heating process was initiated and temperature was maintained at 300 K. To maintain temperature and hydrogen bonds of the systems, the Langevin dynamics [69] and SHAKE algorithm [70] were used, respectively. Then, in the next step, the complexes pressure was maintained for 100 ps to achieve pressure equilibrium using NPT ensemble. The CPPTRAJ module [71] was used to evaluate simulation trajectories.

### 2.7. Estimation of Binding Free Energy

MMPBSA.py module [72] was employed to calculate the solvation and associated binding free energies produced as an outcome of interactions between the vaccine and all three receptors. The binding free energy was calculated on total of 100 frames taken from the molecular dynamics simulation’s trajectories. The equation used to determine the total binding energy for all the docked complexes follows:

ΔG _bind_ = ΔG _bind, vaccum_ + ΔG _solv,_ − (ΔG _solv, vaccine_+ ΔG _solv, receptor_)

### 2.8. In Silico Immune Profiling of Multi-Epitopes Vaccine Construct

Consequently, the C-Immune Simulation server (or C-ImmSim) [73] was used to check the in silico immune profiling of the multi-epitopes vaccine construct. The algorithm used in this server is mainly based on position-specific matrix (PSSM) to evaluate the immunogenic potential of a given antigen. All the input parameters were set as default except number of steps, which were set to 1000 [74].

### 2.9. Codon Adaptation and Cloning

The sequence of vaccine was back-translated to generate DNA sequence and then codon usage was adapted to *E. coli* K-12 strain to achieve high expression of the vaccine. This was done through Java Codon Adaptation Tool (JCat) server [75]. Expression of the construct was assessed by codon adaptation index (CAI) and GC-content. This was followed by in silico cloning of the vaccine in *E. coli* pET28a vector by Snapgene tool.

### 2.10. Disulfide Engineering

The stability of protein was increased by introduction of disulfide bonds [76]. Design 2.0 [77] was used to perform disulfide engineering.

## 3. Results and Discussion

### 3.1. Pan-Proteome Analysis and Prioritization of Potential Vaccine Candidates

**Figure 2:**
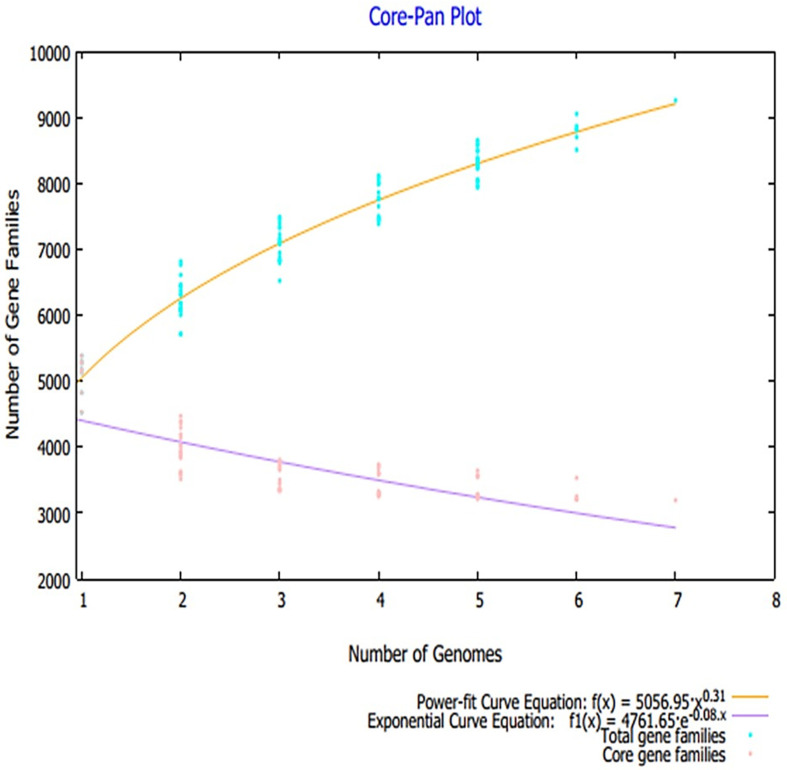
Core–pan plot obtained through pan-proteome analysis.

Advances in sequencing technologies and the field of metagenomics resulted in a paradigm shift in microbial genomics, thus allowing comparison of large scale pan-genomics studies. This further facilitates estimation of genomic diversity and determination of highly conserved core genomes. In vaccine designing, this core genome has broad spectrum applications to select the most conserved antigen for a broad spectrum vaccine design [28,78]. A total number of 973 proteomes were retrieved from the NCBI genome database, the details of which are tabulated in Table S1, and only reference proteome of each *Klebsiella* species was used for pan-proteome analysis because the reference proteome is completely annotated. The core-proteome of the referenced species contains ~22,393 proteins with an average of 3635 proteins for each strain. The core–pan plot total genes and core genes for each analyzed genome are presented in Figure 2. The core proteome was then filtered for several parameters that were set to prioritize potential vaccine candidates.

These parameters can be briefly discussed as nonredundancy check, subcellular localization, homology check against human host, antigenicity check, allergenicity check, virulent analysis, adhesion analysis, physicochemical characterization, and BLASTp check against human probiotic bacteria. First, redundancy check was performed that predicted 5864 proteins as nonredundant and ensured that only one copy of each protein is present in the core genome sequence file [79]. Furthermore, only 43 protein sequences were found secretory and exoproteome in nature. The surface-localized proteins are good candidates for vaccine design [80]. Virulent proteins have been reported to have an important role in the pathogenesis of the organism and play a vital role as a good vaccine candidate [34]. Virulent peptides can provoke immune response properly, so virulent protein analysis was performed in order to predict virulent proteins. Virulent analysis indicated that 19 proteins are virulent and these proteins were used for further analysis. The 19 virulent proteins are tabulated in Table 1. Next, transmembrane helices check and physicochemical analysis was carried out that filtered 18 proteins as thermodynamically stable, have few transmembrane helices, and exhibit good physicochemical properties. To avoid cross reactivity and autoimmune reactions inside the host, all core proteins sequences were subjected to comparative homology analysis, among which 15 proteins were predicted as nonhomologous to humans. From 15 human nonhomologous proteins, 34 proteins were homologues to human normal intestinal flora, which were discarded from the study. Antigenic proteins are important for designing a vaccine as they are the prime component to stimulate host immune responses against any antigen. Five proteins were predicted as virulent and forwarded to additional analysis. Similarly, an allergenicity check was performed to discard proteins that are able to generate allergic reactions. As such proteins are discouraged during vaccine design, they were not picked and only nonallergic protein candidates were opted. Only two proteins were picked as nonallergenic and used in downward analysis. Adhesion analysis was important in part to ensure selection of proteins that are key in pathogen attachment to the host cells and significant for initiating infection pathway [81]. In the past, adhesive proteins were unveiled to be important candidates for vaccine development [82]. Herein, both the proteins were found to play adhesive role and were selected. Lastly, to avoid accidental inhibition of the good bacteria, the filtered proteins were also tested for homology against different probiotic species. Proteins with no significant hits were only selected. The BLASTp results concluded both proteins as nonhomologous to the host probiotic bacteria. These two proteins were TonB-dependent siderophore receptor and siderophore enterobactin receptor FepA.

### 3.2. Mapping of B and T Cell Epitopes

In B and T cell epitope mapping phase, all the prioritized shortlisted proteins sequence were analyzed for epitope prediction. Consequently, three B cell epitopes were predicted for both proteins and predicted B cell epitopes were further screened for B cell derived T cell epitopes, as shown in Table 2. Antigenicity of the predicted epitopes was checked. Only those epitopes were selected whose predicted antigenic prediction score was greater than 0.5 and were considered as good candidates for vaccine development. Three epitopes were predicted as good antigenic epitopes for TonB-dependent siderophore receptor while four epitopes were selected for siderophore enterobactin receptor FepA. The epitopes selected fulfill different good antigenic epitope properties, such as antigenic, soluble, nonallergenic, nontoxic, and virulent. The population coverage of the epitopes is 82.86%. Further details on the population coverage can be found in Table S2.

### 3.3. Multi-Epitopes Vaccine Construct

**Figure 3:**
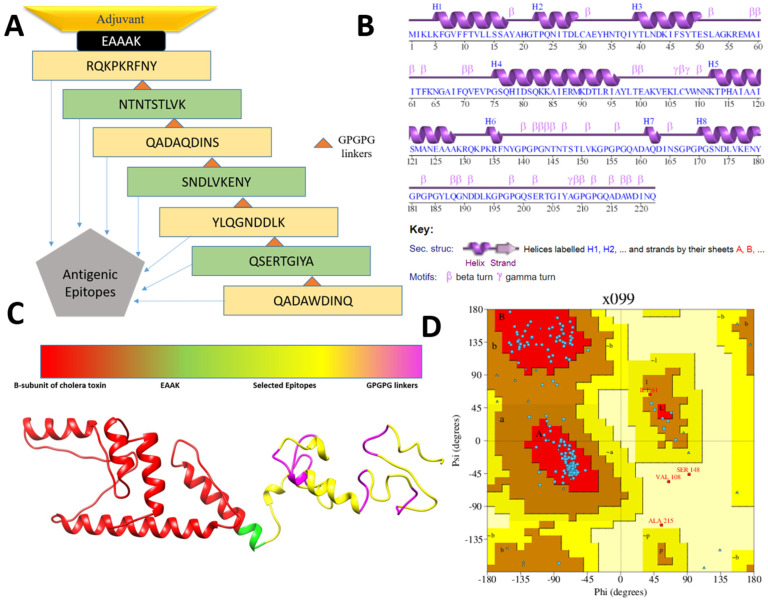
Schematic vaccine design (**A**), secondary structure of the vaccine (**B**), 3D structure of the vaccine (**C**) and Ramachandran plot of the vaccine (**D**).

A multi-epitopes vaccine construct was engineered that comprises different epitopes from the prioritized vaccine proteins and fused in order to generate substantial immune responses. In total, seven epitopes (noted in Table 2) were selected based on their ability to fulfill several different parameters, as presented in Table 2. The selected epitopes are capable of eliciting both humoral- and cell-mediated immunity as the epitopes contain sequences of both B and T cells. In addition, the epitopes are free from allergic sequence, which ensures avoiding allergic reactions. The epitopes are nontoxic and predicted to not cause any toxicity inside the cells. Similarly, the epitopes are water soluble and have high affinity for the most prevalent DRB*0101 alleles, thus providing them with an efficient ability to binding to the host immune receptors [40]. The epitopes are antigenic and vital in binding with the products of immune system. Further, the epitopes are virulent, which ensures the epitopes to activate infectious pathway and allow the host immune system to respond to the antigen. The epitopes were joined via GPGPG linkers that allow easy presentation of the epitopes to the host immunity and keep the epitopes separated [32]. The final epitopes peptide was then joined to an adjuvant molecule to further boost the immune simulation ability of the designed vaccine [83]. The cholera B subunit was considered as the adjuvant molecule herein as the adjuvant is safe to be used in humans and allows active stimulation of cytotoxic T cells, which is a key player in destroying foreign pathogens inside the human body [59,84]. The designed vaccine molecule is 222 amino acid long, spanning epitopes from two vaccine proteins. The designed vaccine construct is schematically presented in Figure 3A.

### 3.4. Three Dimensional Structure Modeling and Processing

The secondary and 3D structure of the vaccine is presented in Figure 3A,B. The 3D structure of the vaccine was modeled ab initio as no appropriate template structure was available for homology-based structure prediction. The plot analysis presented by Ramachandran has shown the residues of the vaccine are in highly preferred zones, with 84.5% in most favored regions, 12.3% in additionally allowed regions, and 2.8% in generously allowed regions (Figure 3D). The ERRAT score of the vaccine is 80.24 and the Verify-3D score is 81.98. From the secondary structure point of view, the vaccine has 36.9% of alpha helix, 5.4% 3–10 helix, and 57.7% of beta turns, gamma turns, and helix–helix interactions. Once the vaccine model was achieved, loop modeling was performed to obtain the most optimal structure. In this regard, the residues-range Gln158-Asp160, Ala161-Ser172, and Pro182-Gly199 loop regions were loop modeled using Galaxyloop. Next, the structure was refined for structural errors. The refined structures along with the initial structure are given in Table 3. The top one structure was selected because of improved galaxy energy of −3648.56 kcal/mol. The structure also has improved residues in the Rama-favored region, i.e., 90.5%. The refined model also has improved rotamer and clash scores.

### 3.5. Physiochemical Analysis of the Vaccine

**Figure 4:**
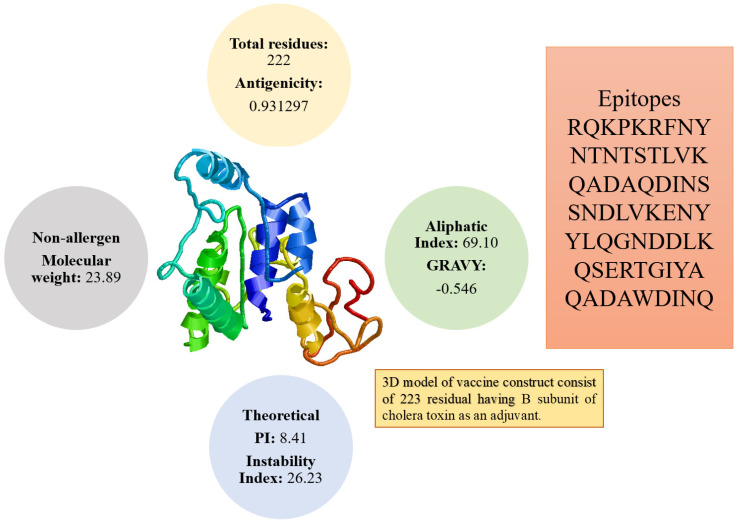
Different physicochemical properties, 3D model, and epitopes sequence of the designed vaccine construct.

The overall weight of the vaccine is 23.89 kDa. This small-size vaccine allows its easy purification during experimental studies. The vaccine construct was found to be positive for antigenicity score of 0.93. In addition, the vaccine construct was further checked for its solubility prediction that revealed good water solubility. The vaccine was further reported to be a nonallergen. Stability index value of the vaccine is 26.23 showing that the vaccine construct is stable. The GRAVY index (−0.546) highlighted that the vaccine hydrophilic. The pI value of the vaccine is 8.41 while its aliphatic index score is 69.10. The physicochemical properties of vaccine are presented in Figure 4.

### 3.6. Interaction Analysis

Molecular docking approach was used to dock the vaccine construct with immune receptors in order to check its binding affinity and presentation ability to the host immune system for robust production of immune responses [85]. The PATCHDOCK solutions are given in Tables S3–S5, respectively. The top 10 best solutions of the vaccine with TLR4, MHC-I, and MHC-II are tabulated in Table 4.

**Figure 5:**
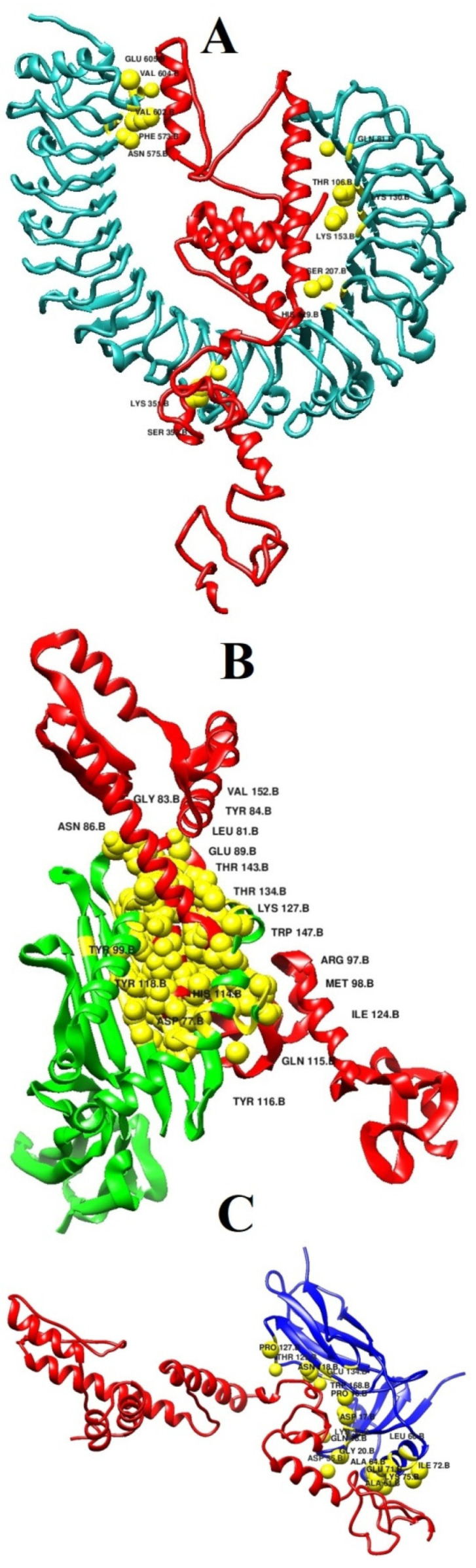
Docked binding mode and interactions of the vaccine with respect to immune receptors. (**A**) vaccine-TLR4, (**B**) vaccine-MHC-I, and (**C**) vaccine-MHC-II.

For each complex, the best solution in term of global energy was selected. For TLR4, solution 4 of PATCHDOCK was selected as the best complex as its global binding energy score is −16.01 kcal/mol, which can be split into −31.00 kcal/mol from attractive van der Waals energy, 8.99 kcal/mol from repulsive van der Waals energy, 10.72 kcal/mol from atomic energy contact, and −4.13 kcal/mol from hydrogen bond energy. The vaccine was disclosed to form multiple hydrogen bonds with the TLR4 receptor via interaction with Gln81, Thr106, Lys130, Lys153, Ser207, His229, Lys351, Ser352, Phe573, Asn575, Val602, Val604, and Glu605 (Figure 5A). Similarly, in the case of MHC-I, solution 7 was selected because of global energy of −2.22 kcal/mol, with contribution of −2.06 from attractive van der Waal energy, 0.04 kcal/mol from repulsive van der Waals energy, 0.98 kcal/mol from atomic contact energy, and −0.50 kcal/mol energy from hydrogen bond. The following residues of the MHC-I interact through hydrogen bond with Asp77, Leu81, Gly83, Tyr84, Asn86, Glu89, Arg97, Met98, Tyr99, His114, Gln115, Tyr116, Tyr118, Ile124, Lys127, Thr134, Thr143, Trp147, and Val152 (Figure 5B). Solution 7 was selected for MHC-II. The global energy of this complex is −39.32 kcal/mol. The vaccine interacts with Pro16, Asp17, Gln18, Gly20, Asp35, Lys39, Ala64, Leu66, Glu71, Ile72, Lys75, and Ala61. Asn118, Pro127, Thr129, and Glu134 (Figure 5C).

### 3.7. Molecular Dynamics Simulation Analysis

**Figure 6:**
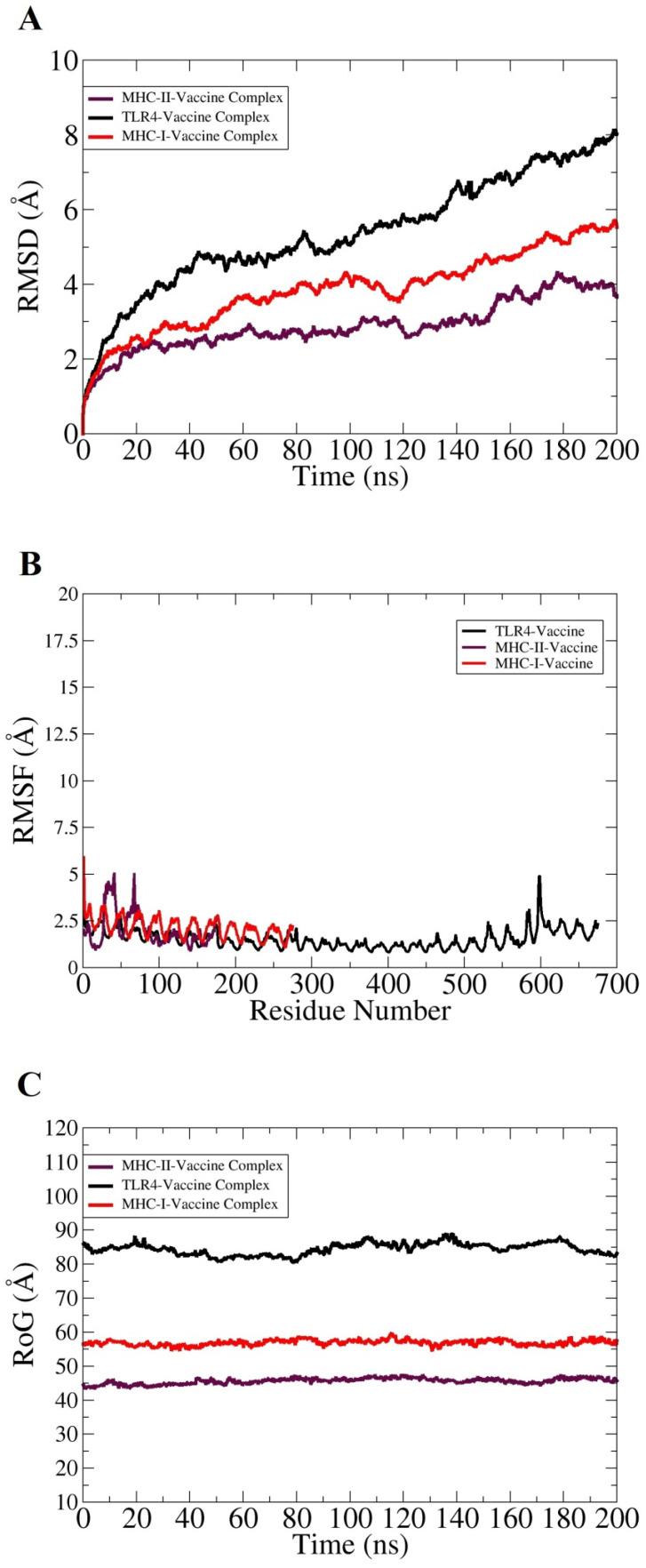
Structural stability analysis of complexes based on carbon alpha atoms: (**A**) RMSD, (**B**) RMSF, and (**C**) RoG.

Taking into account the effectivity of molecular dynamics simulation in validating complexes dynamics and stability, 200 ns long all atom MD simulations was performed for the best docked vaccine and immune receptors complex. This analysis was essential for understanding the valuable information regarding the system’s dynamics and to shed light on key vaccine binding interactions with the receptors. The trajectories of MD simulations were evaluated for different structural parameters such as root mean square deviations (RMSD) [86], root mean square fluctuations (RMSF) [87], and radius of gyrations (RoG) [88]. All these analyses were carried out considering carbon alpha atoms of the systems. RMSD was calculated first for all systems to decipher the extent of deviations the complexes can experience, considering the initial complex conformation as a reference. Higher RMSD is the output of significant instability and can be correlated to conformational changes of the investigated system. As depicted in Figure 6A, the systems are undergoing consistent conformational changes as the time proceeds; however, the changes are not sudden and minor regarding the docked conformation. These changes are within the limits and are not affecting overall intermolecular binding and interactions. The inspection of the steady RMSD increase reveal large and complexity of the systems and the presence of large number of loops that is flexible in nature and more dynamic. The TLR4 vaccine complex was found to show high RMSD compared to the MHC-I and MHC-II vaccine complexes. The maximum RMSD reached by the systems were as follows: TLR4 vaccine complex (8 Å), MHC-I vaccine complex (5 Å), and MHC-II vaccine complex (4 Å). Further insights were gained by knowing residue stability of the docked site. For this purpose, the systems RMSDs were split as per residue. The majority of the interacting receptor residues with the vaccine molecule unraveled a highly stable nature, as can be interpreted by the lower RMSF value shown in Figure 6B. The terminal residues in contrast to the core residues were found to have more fluctuations, which are expected due to the flexible nature of the biomolecule terminals. Overall, the average RMSF of the systems is <3 Å, which indicates formation of highly stable complexes and good affinity of the vaccine molecule for the receptors. Lastly, conformational equilibrium of the complexes was evaluated by RoG analysis. Lower RoG value implies a tight packing of the complexes while a higher value implies and corresponds to a loose packing of system’s atoms. The RoG analysis results were in line with that of RMSD (Figure 6C). The TLR4 vaccine complex was reported to produce a higher RoG value, which is obvious because of the heavy nature of the complex. The highest RoG for the TLR4 vaccine complex reaches 90 Å, while for the MHC-I and MHC-II vaccine complexes the maximum RoG value is 60 and 45 Å, respectively.

### 3.8. Calculation of Binding Free Energies

The MMGB/PBSA method is a popular technique and commonly employed to accurately predict the binding free energies of complexes [89]. This method is less computationally expensive and more productive than most molecular docking approaches. MMGB/PBSA infers net binding free energy, where negative binding free energy is an indication of high receptor–ligand affinity, while positive net binding energy demonstrates low-docked stability. It was estimated that the TLR4 and MHC-I vaccine complexes report that both gas phase and solvation energy highly dominate the intermolecular interactions. In particular, the van der Waals energy, followed by electrostatic energy, was favorable in complex formation. Likewise, the polar solvation energy showed significant domination while nonpolar solvation energy is also supporting complex formation. The net binding free energy of complexes greatly supports intermolecular affinity by securing energy values of −714.28 kcal/mol (MMGBSA) and −678.52 kcal/mol (MMPBSA) for the TLR4 vaccine complex and −610.69 kcal/mol (MMGBSA) and −649.36 kcal/mol (MMPBSA) for the MHC-I vaccine complex. For the MHC-II vaccine complex, the van der Waals energy played a significant role in binding affinity while electrostatic energy is nonsignificant. The polar and nonpolar solvation energy demonstrates favorable contribution to intermolecular affinity. The net binding energy of the MHC-II vaccine complex is −73.29 kcal/mol in case of MMGBSA and −74.82 kcal/mol in MMPBSA. The contribution of each energy term in MMGBSA and MMPBSA for complexes is tabulated in Table 5.

### 3.9. In Silico Expression Analysis of Multi-Epitope Vaccine Construct

**Figure 7:**
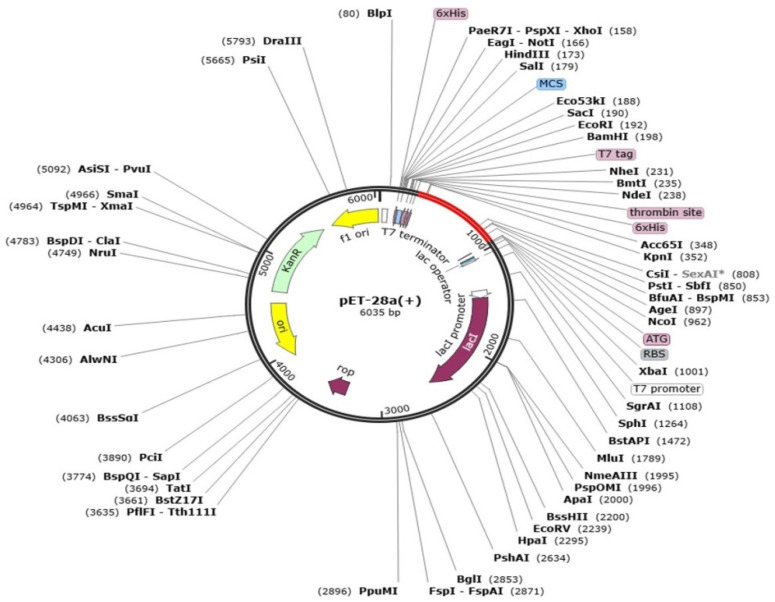
Cloned vaccine sequence (shown in red) into pET-28a(+) expression vector (shown in black).

Codon optimization defines genetic engineering methodologies that optimize codon to assure a maximum level of targeted protein production and expression. Before codon optimization, the peptide sequence of the final vaccine construct was reverse transcribed to DNA sequence for codon optimization by using the java codon optimization tool (JCat tool), and the expression system was investigated by CAI (codon adaptation index) and GC content of construct. The CAI value of the vaccine is 0.96 and GC content is 52.4%. The CAI value is considered good and the vaccine sequence can be inferred to have good expression rate. Lastly, the optimized vaccine construct was cloned into pET-28a(+) and the expression vector, as shown in Figure 7.

### 3.10. Immune Simulation

**Figure 8:**
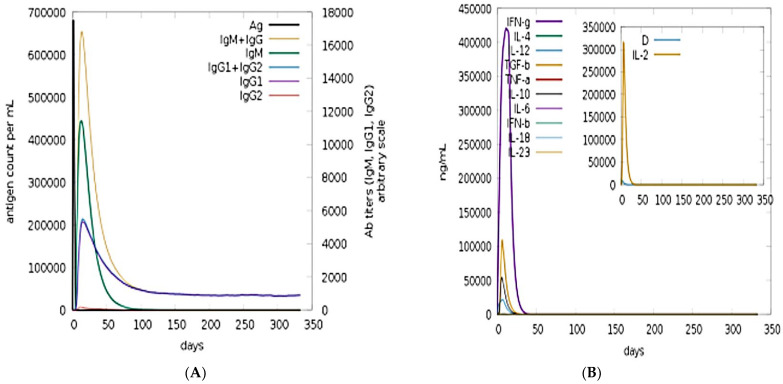
Immune simulations in response to the vaccine antigen: (**A**) antibodies count and (**B**) interleukins and cytokines counts in response to the antigen.

C-Immune simulation was conducted in order to predict putative immune responses of the model vaccine construct. Both primary and secondary responses with respect to days and major immune players are shown in Figure 8A. Robust level of IgM and IgG can be seen, and combined IgM and IgG ratios reached 25,000 counts/mL. Among the interleukins and cytokines, the IFN-g was found in high concentration in response to the antigen (Figure 8B).

### 3.11. Disulfide Engineering of the Vaccine

**Figure 9:**
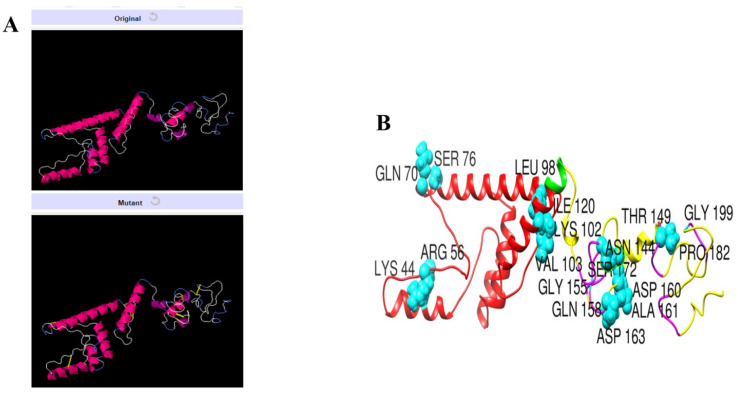
Disulfide engineering of the designed vaccine construct. (**A**) Wild and mutant vaccine. The introduced disulfide bonds are shown by yellow sticks. (**B**) Highlighted mutated residues of the vaccine structure.

Disulfide engineering is considered an important tool and used extensively in improving protein stability and modifying functional characteristics. The designed vaccine construct was subjected to disulfide engineering to highlight less stable regions of the vaccine and improve it for future applications. Concerning the nine residue pairs (Lys44-Arg56, Gln70-Ser76, Leu98-Lys102, Val103-Ile120, Asn144-Thr149, Gly155-Asp163, Gln158-Asp160, Ala161-Ser172, and Pro182-Gly199), the binding energy is an important contributor to vaccine instability and was high for all the residues (>2 kcal/mol). The wild and mutant structure of the vaccine is presented in Figure 9.

## 4. Conclusions

*Klebsiella* species are responsible for variety of infections, including life threatening respiratory, bloodstream, and liver infections. Since no FDA licensed vaccine is available for preventing *Klebsiella* infections, there is an urgent need to propose novel vaccine candidates to be experimentally tested for immune protection efficacy. The traditional vaccine design strategy is expensive and very slow. Computational approaches in this regard can accelerate vaccine development process by providing ideal vaccine candidates against *Klebsiella*. In this present study, we applied a computational vaccine design approach on all reference genome of *Klebsiella* genus species and as such identified two highly conserved and potential vaccine candidates that fulfilled several vaccine candidacy parameters. These proteins are TonB-dependent siderophore receptor and siderophore enterobactin receptor FepA. Both proteins were subjected to epitope mapping phase where only seven epitopes were highly ranked and used in a multi-epitope peptide vaccine construction. Further, the designed vaccine ensemble revealed robust interaction energy and has a stable binding conformation with the tested immune receptor. The application of computer aided vaccine design can significantly reduce the vaccine development cost and in less time. The process can also aid in identifying the correct vaccine candidates for experimental evaluations. The results of the study are promising and must be evaluated experimentally to validate biological effectiveness of the designed vaccine construct.

## Figures and Tables

**Table 1:** Different set of parameters used to prioritize potential vaccine candidates. In the table, only virulent proteins are shown along with the value for all the vaccine parameters.

Proteins	Transmembrane Helices	Physiochemical Properties									NCBI Blast(Human)			Antigenisity	Adhesion	Allergenisity	NCBI Blast(L. Rhamnosus)			NCBI Blast(L. Casei)			NCBI Blast(L. L. Johnsonii)		
	TMHMM	No of Residues	MW	Theoretical PI	Negatively Charged Residues	Positively Charged Residues	Gravy	Aliphatic Index	Instability Index	Stability	Coverage	Id	E-value	Vexijen/0.7	Vaxign	-	Coverage	Id	E-Value	Coverage	Id	E-Value	Covarge	Id	E-Value
>core/178/1/Org1_Gene2817	0	871	94,813.23	6.36	76	71	−0.385	74.71	27.91	Stability	no similarity	no similarity	no similarity	0.6441	-	-	-	-	-	-	-	-	-	-	-
>core/538/1/Org1_Gene4990	0	618	68,660.6	5.2	71	57	−0.58	69.72	26.43	Stability	no similarity	no similarity	no similarity	0.6041	-	-	-	-	-	-	-	-	-	-	-
>core/644/1/Org1_Gene1920	0	581	62,110.64	5.85	66	58	−0.22	81.79	27.91	Stability	88%	33.27%	1.00E-80	-	-	-	-	-	-	-	-	-	-	-	-
>core/870/1/Org1_Gene3696	0	528	55,681.97	5.9	51	39	−0.071	91.27	42.67	unstable	-	-	-	-	-	-	-	-	-	-	-	-	-	-	-
>core/5642/1/Org1_Gene690	0	206	22,970.88	6.23	25	22	−0.386	77.38	31.68	Stability	100%	43.60%	5.00E-56	-	-	-	-	-	-	-	-	-	-	-	-
>core/5897/1/Org1_Gene256	0	193	21,147.69	5.76	19	14	−0.141	78.08	22.96	Stability	100%	43.14%	2.00E-47	-	-	-	-	-	-	-	-	-	-	-	-
>core/6413/1/Org1_Gene5320	0	161	16,537.39	5.15	10	8	0.096	89.69	32.37	Stability	no similarity	no similarity	no similarity	0.9532	0.965	ALLERGEN	-	-	-	-	-	-	-	-	-
>core/178/3/Org3_Gene3169	0	878	94,683.95	6.04	75	68	−0.366	75.02	24.93	Stability	no similarity	no similarity	no similarity	0.6563	-	-	-	-	-	-	-	-	-	-	-
>core/276/3/Org3_Gene2971	0	789	86,600.87	5.41	81	66	−0.583	60.04	31.03	Stability	no similarity	no similarity	no similarity	0.6852	-	-	-	-	-	-	-	-	-	-	-
>core/325/3/Org3_Gene4813	0	753	82,490.82	5.72	75	62	−0.573	66.99	29.48	Stability	no similarity	no similarity	no similarity	0.8125	0.856	NONALLERGEN	no similarity	no similarity	no similarity	no similarity	no similarity	no similarity	no similarity	no similarity	no similarity
>core/538/3/Org3_Gene4136	0	618	68,572.26	5.07	69	51	−0.586	69.72	25.21	Stability	no similarity	no similarity	no similarity	0.625	-	-	-	-	-	-	-	-	-	-	-
>core/870/3/Org3_Gene994	0	527	55,704	5.75	53	40	−0.089	89.39	37.5	Stability	95%	27.03%	2.00E-21	0.4053	-	-	-	-	-	-	-	-	-	-	-
>core/538/4/Org4_Gene3945	0	622	69,756.5	5.22	71	56	−0.668	64.55	25.01	Stability	no similarity	no similarity	no similarity	0.5795	-	-	-	-	-	-	-	-	-	-	-
>core/3258/4/Org4_Gene4637	0	300	31,253.99	9.01	10	15	0.064	85.13	31.98	Stability	no similarity	no similarity	no similarity	0.7627	0.908	ALLERGEN	-	-	-	-	-	-	-	-	-
>core/6413/4/Org4_Gene2075	0	167	17,112.08	5.03	9	7	0.095	84.73	23.68	Stability	no similarity	no similarity	no similarity	0.8816	0.959	ALLERGEN	-	-	-	-	-	-	-	-	-
>core/591/6/Org6_Gene4047	0	597	61,743.78	7.64	37	38	−0.123	85.28	15.68	Stability	no similarity	no similarity	no similarity	0.6161	-	ALLERGEN									
>core/325/7/Org7_Gene2597	0	742	82,442.17	5.42	86	73	−0.68	64.78	33.5	Stability	no similarity	no similarity	no similarity	0.8217	0.807	NONALLERGEN	no similarity	no similarity	no similarity	no similarity	no similarity	no similarity	no similarity	no similarity	no similarity
>core/538/7/Org7_Gene4577	0	615	68,043.77	5.17	66	51	−0.481	70.99	26.7	Stability	no similarity	no similarity	no similarity	0.5512	-	ALLERGEN	-	-	-	-	-	-	-	-	-
>core/870/7/Org7_Gene4277	0	527	56,010.35	6.05	49	40	−0.117	89.73	41.54	unstable	-	-	-	-	-	-	-	-	-	-	-	-	-	-	-

**Table 2:** Final set of selected epitopes that were filtered based on several parameters.

Proteins	B-Cell Epitopes	T-Cell Epitopes	Percentile Rank	MHC II	Percentile Rank	Common Peptides	Antigenicity/0.5		Allergenicity	Solubility	MHC Pred	Toxicity	Virulent	IFN Gamma	Final
>core/325/3/Org3_Gene4813 (TonB-dependent siderophore receptor)	YGRQKPKRFNYKGESVSGSELNEV	RQKPKRFNY	1.3	RQKPKRFNYKGESVS	54	RQKPKRFNY	0.9784	Antigen	nonallergen	Good water solubility	2.37	nontoxin	Virulent	positive	Selected
	YSRQGNLYAGDTQNTNTSTLVKSMYGKETNRLY	NTNTSTLVK	2.3	QNTNTSTLVKSMYGK	55	NTNTSTLVK	0.7856	Antigen	nonallergen	Good water solubility	55.98	nontoxin	Virulent	positive	Selected
	SKTQADAQDINSGHEAARTGSYAGSYPAGREGVVNKDIHG	QADAQDINS	7.5	QADAQDINSGHEAAR	85	QADAQDINS	1.4865	Antigen	nonallergen	Good water solubility	4.83	nontoxin	Virulent	positive	Selected
>core/325/7/Org7_Gene2597 (siderophore enterobactin receptor FepA)	SRQGNLYAGDTQNTNSNDLVKENYGKETNRLYR	SNDLVKENY	0.24	SNDLVKENYGKETNRLYR	59	SNDLVKENY	0.8435	Antigen	nonallergen	Good water solubility	46.99	nontoxin	Virulent	positive	Selected
	QTNPNYILYSKGQGCYASKSGCYLQGNDDLKAE	YLQGNDDLK	3.4	GCYLQGNDDLKA	41	YLQGNDDLK	1.4275	Antigen	nonallergen	Good water solubility	21.73	nontoxin	Virulent	positive	Selected
	LDKTQADAWDINQGHQSERTGIYADTLPAGREGVE	QSERTGIYA	1.7	QSERTGIYADTL	55	QSERTGIYA	0.5139	Antigen	nonallergen	Good water solubility	33.19	nontoxin	Virulent	positive	Selected
		QADAWDINQ	4.5	QADAWDINQGHQS	71	QADAWDINQ	0.8036	Antigen	nonallergen	Good water solubility	10.09	nontoxin	Virulent	positive	Selected

**Table 3:** Refined vaccine models together with different parameters. The refined models are ranked on basis of galaxy energy.

Model	RMSD	MolProbity	Clash Score	Poor Rotamers	Rama Favored	GALAXY Energy
Initial	0	3.91	109.2	9.7	84.5	24,937.41
MODEL 1	0.89	1.48	1.9	0.6	90.5	−3648.56
MODEL 2	1.29	1.56	3	0	92.3	−3646.33
MODEL 3	0.88	1.50	2.4	0	92.3	−3638.63
MODEL 4	0.88	1.35	1.4	0	91.8	−3624.25
MODEL 5	0.95	1.47	1.9	0	90.9	−3623.95
MODEL 6	0.85	1.56	2.4	0	90.5	−3618.86
MODEL 7	1.65	1.57	3	0	91.8	−3617.76
MODEL 8	2.75	1.62	3.2	0	91.4	−3617.33
MODEL 9	1.45	1.51	2.7	0	92.7	−3616.55
MODEL 10	1.05	1.66	3.5	0.6	90.9	−3616.14

**Table 4:** Refined docked solutions of vaccine with TLR4, MHC-I, and MHC-II. All values are given in kcal/mol.

TLR4						
Rank	Solution Number	Global Energy	Attractive van der Waals Energy	Repulsive van der Waals Energy	Atomic Contact Energy	Hydrogen Bonds Energy
1	4	−16.01	−31.00	8.99	10.72	−4.13
2	2	−8.24	−30.86	13.13	15.55	−4.23
3	1	−3.81	−33.73	10.52	21.45	−6.01
4	10	4.69	−45.43	21.19	20.21	−1.33
5	5	8.46	−24.26	14.75	19.26	−1.50
6	9	10.47	−0.37	0.00	1.03	0.00
7	6	17.68	−18.48	22.97	8.59	−1.86
8	7	23.80	−20.71	10.74	11.10	−0.81
9	3	31.16	−12.53	25.41	11.03	−0.30
10	8	44.88	−21.53	7.66	10.71	−2.45
**MHC-I**						
**Rank**	**Solution Number**	**Global Energy**	**Attractive van der Waals Energy**	**Repulsive van der Waals Energy**	**Atomic Contact Energy**	**Hydrogen Bonds Energy**
1	7	−2.22	−2.06	0.04	0.98	−0.50
2	9	12.40	−0.55	0.00	0.34	0.00
3	8	13.34	−31.79	53.87	6.76	−5.68
4	4	48.19	−31.31	94.84	6.68	−8.33
5	2	603.03	−55.26	811.32	5.43	−5.36
6	10	654.52	−25.48	850.38	7.08	−5.82
7	5	697.54	−49.00	968.22	−2.34	−3.14
8	3	1338.16	−36.09	1705.36	9.00	−6.47
9	1	1394.24	−29.34	1801.68	−2.37	−1.68
10	6	4166.32	−67.09	5348.85	−7.31	−7.27
**MHC−II**						
**Rank**	**Solution Number**	**Global Energy**	**Attractive van der Waals Energy**	**Repulsive van der Waals Energy**	**Atomic Contact Energy**	**Hydrogen Bonds Energy**
1	7	−39.32	−35.52	14.84	6.51	−2.57
2	9	−32.36	−28.35	9.77	−1.69	−0.95
3	4	−25.80	−29.69	12.42	15.73	−4.94
4	2	−18.97	−41.15	34.17	3.31	−5.99
5	1	−9.05	−7.99	1.86	1.04	−0.31
6	8	3.14	−13.88	1.90	6.13	−0.67
7	10	7.17	−22.42	45.49	−0.73	−1.11
8	3	13.32	−0.85	0.00	−0.73	0.00
9	5	13.70	−2.59	1.00	2.27	−0.41
10	6	1319.04	−56.48	1760.85	6.51	−6.93

**Table 5:** MMGB/PBSA binding free energies of receptor(s)–vaccine complex. All values are given in kcal/mol.

MMGBSA		MMPBSA	
**TLR4 Vaccine Complex**			
Energy Component	Average	Energy Component	Average
VDWALLS	−324.47	VDWALLS	−324.47
EEL	−172.48	EEL	−172.48
EGB	−198.69	EPB	−159.57
ESURF	−18.64	ENPOLAR	−22.00
Delta G gas	−496.95	Delta G gas	−496.95
Delta G solve	−217.33	Delta G solve	−181.57
Total	−714.28	Total	−678.52
**MHC-I Vaccine Complex**			
Energy Component	Average	Energy Component	Average
VDWALLS	−311.42	VDWALLS	−311.42
EEL	−172.58	EEL	−172.58
EGB	−105.66	EPB	−147.83
ESURF	−21.03	ENPOLAR	−17.53
Delta G gas	−484	Delta G gas	−484
Delta G solve	−126.69	Delta G solve	−165.36
Total	−610.69	Total	−649.36
**MHC-II Vaccine Complex**			
Energy Component	Average	Energy Component	Average
VDWALLS	−80.35	VDWALLS	−80.35
EEL	90.16	EEL	90.16
EGB	−67.33	EPB	−73.09
ESURF	−15.77	ENPOLAR	−11.54
Delta G gas	9.81	Delta G gas	9.81
Delta G solve	−83.1	Delta G solve	−84.63
Total	−73.29	Total	−74.82

## Data Availability

The data presented in this study are available within the article.

## Supplementary Materials

The following are available online at https://www.mdpi.com/article/10.3390/vaccines9101087/s1. Table S1. Statistics of species; Table S2. Population coverage statistics; Table S3. Results of multi-epitope vaccine binding with TLR4; Table S4. Results of multi-epitope vaccine binding with MHCI; Table S5. Results of multi-epitope vaccine binding with MHCII.
