# Supplementary material for: A Comprehensive Computer Aided Vaccine Design Approach to Propose a Multi-Epitopes Subunit Vaccine against Genus Klebsiella Using Pan-Genomics, Reverse Vaccinology, and Biophysical Techniques
Source: Vaccines (Basel). 2021 Sep 27;9(10):1087. doi: 10.3390/vaccines9101087 (PMC8540426; doi:10.3390/vaccines9101087)
Supplement: Supplementary file 1 [file vaccines-09-01087-s001.zip › S-Table S1.pdf]

**SPECIES**	**REPORTED**	**Complete Sequenced**
K. aerogenes	363	32
K. oxytoca	222	32
K. michiganansis	296	27
K. pnemoniae	11143	764
K. quasipneumonia	646	55
K. grimontii	110	15
K. variicola	495	48
