# Supplementary material for: A Comprehensive Computer Aided Vaccine Design Approach to Propose a Multi-Epitopes Subunit Vaccine against Genus Klebsiella Using Pan-Genomics, Reverse Vaccinology, and Biophysical Techniques
Source: Vaccines (Basel). 2021 Sep 27;9(10):1087. doi: 10.3390/vaccines9101087 (PMC8540426; doi:10.3390/vaccines9101087)
Supplement: Supplementary file 1 [file vaccines-09-01087-s001.zip › S-Table S2.pdf]

population/area	Class combined		
	coverage[a]	average_hit[b]	pc90[c]
Algeria	77.15%	6.96	3.06
Algeria Arab	77.15%	6.96	3.06
American Samoa	98.75%	14.16	9.67
American Samoa Polynesian	98.75%	14.16	9.67
Argentina	99.07%	17.68	11.57
Argentina Amerindian	98.64%	15.91	10.12
Argentina Caucasoid	80.65%	7.52	3.62
Australia	96.33%	15.34	8.99
Australia Australian Aborigines	91.52%	12	7.3
Australia Caucasoid	99.94%	21.31	16.49
Austria	99.99%	29.89	23.29
Austria Caucasoid	99.99%	29.89	23.29
Belarus	43.81%	3.28	1.25
Belarus Caucasoid	43.81%	3.28	1.25
Belgium	99.87%	22.8	16.22
Belgium Caucasoid	99.87%	22.8	16.22
Bolivia	77.82%	6.51	3.16
Bolivia Amerindian	77.82%	6.51	3.16
Borneo	49.02%	3.78	1.37
Borneo Austronesian	49.02%	3.78	1.37
Brazil	98.59%	19.04	12.17
Brazil Amerindian	96.52%	13.22	8.55
Brazil Caucasoid	99.89%	25.33	18.24
Brazil Mixed	99.33%	22.03	14.96
Brazil Mulatto	74.09%	6.66	2.7
Bulgaria	99.75%	21.26	15.07
Bulgaria Caucasoid	99.79%	21.49	15.24
Bulgaria Other	99.58%	14.45	10.05
Burkina Faso	67.18%	6.47	2.13
Burkina Faso Black	67.18%	6.47	2.13
Cameroon	94.85%	15.63	8.59
Cameroon Black	94.85%	15.63	8.59
Canada	38.41%	2.95	1.14
Canada Amerindian	38.41%	2.95	1.14
Cape Verde	99.84%	25.64	18.29
Cape Verde Black	99.84%	25.64	18.29
Central Africa	94.79%	15.77	8.59
Central African Republic	87.54%	9.62	5.62
Central African Republic Black	87.54%	9.62	5.62
Central America	53.80%	4.48	1.52
Chile	98.66%	19.18	12.36

Chile Amerindian	99.90%	21.46	15.3
Chile Mixed	95.31%	15.5	8.7
China	97.83%	17.65	10.56
China Oriental	97.83%	17.65	10.56
Colombia	57.86%	4.98	1.66
Colombia Amerindian	47.40%	3.71	1.33
Colombia Black	66.51%	5.87	2.09
Colombia Mestizo	62.45%	5.58	1.86
Congo	68.66%	5.93	2.23
Congo Black	68.66%	5.93	2.23
Cook Islands	78.59%	7.11	3.27
Cook Islands Polynesian	78.59%	7.11	3.27
Costa Rica	24.31%	1.78	0.92
Costa Rica Mestizo	24.31%	1.78	0.92
Croatia	99.93%	24.28	17.34
Croatia Caucasoid	99.93%	24.28	17.34
Cuba	99.84%	25.46	18.21
Cuba Caucasoid	99.05%	17.32	12.16
Cuba Mixed	85.48%	8.03	4.82
Cuba Mulatto	98.74%	17.46	11.97
Czech Republic	99.87%	25.84	18.64
Czech Republic Caucasoid	99.90%	26.3	19.34
Czech Republic Other	64.14%	5.34	1.95
Denmark	88.98%	8.7	6.35
Denmark Caucasoid	88.98%	8.7	6.35
East Africa	97.08%	17.6	10.15
East Asia	99.67%	23.36	16.07
Ecuador	89.16%	10.12	6.46
Ecuador Amerindian	89.16%	10.12	6.46
England	100.00%	30.89	24.31
England Caucasoid	100.00%	30.89	24.31
Equatorial Guinea	47.58%	3.76	1.34
Equatorial Guinea Black	47.58%	3.76	1.34
Ethiopia	83.00%	7.63	4.12
Ethiopia Black	83.00%	7.63	4.12
Europe	99.96%	27.12	20.94
Fiji	79.87%	6.88	3.48
Fiji Melanesian	79.87%	6.88	3.48
Finland	100.00%	25.53	19.81
Finland Caucasoid	100.00%	25.53	19.81
France	99.98%	28.1	21.73
France Caucasoid	99.98%	28.1	21.73
Gabon	41.78%	3.15	1.2

Gabon Black	41.78%	3.15	1.2
Georgia	99.58%	22.46	15.47
Georgia Caucasoid	99.74%	23.21	16.11
Georgia Kurd	98.19%	15.61	10.2
Germany	99.99%	30	23.33
Germany Caucasoid	99.99%	30	23.33
Greece	66.92%	5.76	2.12
Greece Caucasoid	66.92%	5.76	2.12
Guatemala	53.11%	4.31	1.49
Guatemala Amerindian	53.11%	4.31	1.49
Guinea-Bissau	98.96%	21.1	14.25
Guinea-Bissau Black	98.96%	21.1	14.25
Hong Kong	96.05%	13.36	8.48
Hong Kong Oriental	96.05%	13.36	8.48
India	97.35%	17.74	10.41
India Asian	97.35%	17.74	10.41
Indonesia	93.11%	13.99	7.82
Indonesia Austronesian	93.11%	13.99	7.82
Iran	99.11%	20.74	14.21
Iran Kurd	55.78%	4.53	1.58
Iran Persian	99.14%	20.9	14.31
Ireland Northern	100.00%	31.45	25.12
Ireland Northern Caucasoid	100.00%	31.45	25.12
Ireland South	100.00%	30.67	24.15
Ireland South Caucasoid	100.00%	30.67	24.15
Israel	96.69%	16.82	9.67
Israel Arab	98.19%	18.8	11.58
Israel Jew	98.10%	18.16	11.18
Italy	99.86%	25.24	18.28
Italy Caucasoid	99.86%	25.24	18.28
Ivory Coast	67.75%	5.63	2.17
Ivory Coast Black	67.75%	5.63	2.17
Jamaica	27.41%	2.02	0.96
Jamaica Black	27.41%	2.02	0.96
Japan	99.66%	22.67	15.61
Japan Oriental	99.66%	22.67	15.61
Jordan	95.68%	15.21	8.78
Jordan Arab	95.68%	15.21	8.78
Kenya	89.56%	11.29	6.7
Kenya Black	89.56%	11.29	6.7
Kiribati	10.89%	0.77	0.79
Kiribati Micronesian	10.89%	0.77	0.79
Korea; South	99.74%	24.36	16.89

Korea; South Oriental	99.74%	24.36	16.89
Lebanon	70.46%	6.11	2.37
Lebanon Arab	70.46%	6.11	2.37
Macedonia	75.47%	7.73	2.85
Macedonia Caucasoid	75.47%	7.73	2.85
Malaysia	92.18%	13.45	7.54
Malaysia Austronesian	83.57%	10.42	4.26
Malaysia Oriental	96.39%	15.96	9.27
Mali	96.02%	14.09	8.71
Mali Black	96.02%	14.09	8.71
Martinique	80.26%	8.3	3.55
Martinique Black	80.26%	8.3	3.55
Mexico	99.09%	18.41	12.21
Mexico Amerindian	99.93%	18.81	14.24
Mexico Mestizo	99.41%	20.47	14.37
Mongolia	99.15%	18.12	13.24
Mongolia Oriental	99.15%	18.12	13.24
Morocco	99.77%	24.1	16.88
Morocco Arab	99.90%	25.51	18.59
Morocco Caucasoid	99.58%	22.67	15.63
Nauru	38.66%	2.94	1.14
Nauru Micronesian	38.66%	2.94	1.14
Netherlands	83.44%	7.97	4.23
Netherlands Caucasoid	83.44%	7.97	4.23
New Caledonia	99.66%	18.58	14.06
New Caledonia Melanesian	99.66%	18.58	14.06
New Zealand	84.46%	7.98	4.5
New Zealand Polynesian	84.46%	7.98	4.5
Niue	77.82%	6.66	3.16
Niue Polynesian	77.82%	6.66	3.16
North Africa	99.01%	20.66	14.15
North America	99.89%	25.87	18.77
Northeast Asia	97.88%	17.74	10.64
Norway	94.71%	10.13	7.74
Norway Caucasoid	94.71%	10.13	7.74
Oceania	97.88%	16.61	10.06
Oman	99.69%	18.3	14.3
Oman Arab	99.69%	18.3	14.3
Pakistan	97.13%	11.22	8.44
Pakistan Asian	96.79%	11.13	8.34
Pakistan Mixed	97.73%	11.3	8.59
Papua New Guinea	99.36%	16.25	10.79
Papua New Guinea Melanesian	99.36%	16.25	10.79

Paraguay	4.90%	0.34	0.74
Paraguay Amerindian	4.90%	0.34	0.74
Peru	100.00%	17.59	12.52
Peru Amerindian	100.00%	17.59	12.52
Philippines	96.41%	13.13	8.4
Philippines Austronesian	96.41%	13.13	8.4
Poland	99.96%	26.9	20.69
Poland Caucasoid	99.96%	26.9	20.69
Portugal	99.72%	23.69	16.37
Portugal Caucasoid	99.72%	23.69	16.37
Romania	99.67%	17.97	14.04
Romania Caucasoid	99.67%	17.97	14.04
Russia	99.84%	23.91	16.76
Russia Caucasoid	88.97%	9.13	6.35
Russia Mixed	5.05%	0.35	0.74
Russia Other	100.00%	25.72	19.83
Russia Siberian	99.88%	25.13	17.88
Rwanda	72.05%	7.1	2.5
Rwanda Black	72.05%	7.1	2.5
Samoa	80.86%	7.42	3.66
Samoa Polynesian	80.86%	7.42	3.66
Sao Tome and Principe	99.00%	21.19	14.34
Sao Tome and Principe Black	99.00%	21.19	14.34
Saudi Arabia	99.65%	22.75	15.82
Saudi Arabia Arab	99.65%	22.75	15.82
Scotland	96.82%	15.31	9.21
Scotland Caucasoid	96.82%	15.31	9.21
Senegal	96.92%	16.13	9.53
Senegal Black	96.92%	16.13	9.53
Serbia	73.37%	6.52	2.63
Serbia Caucasoid	73.37%	6.52	2.63
Singapore	97.49%	17.58	10.36
Singapore Austronesian	96.77%	16.94	9.72
Singapore Oriental	94.81%	12.86	8.1
Slovakia	18.28%	1.28	0.86
Slovakia Caucasoid	18.28%	1.28	0.86
Slovenia	84.85%	8.16	4.62
Slovenia Caucasoid	84.85%	8.16	4.62
South Africa	95.27%	14.82	8.57
South Africa Black	94.54%	14.54	8.3
South Africa Other	97.61%	14.73	9.41
South America	95.15%	15.07	8.58
South Asia	98.70%	19.46	12.86

Southeast Asia	97.66%	17.31	10.29
Southwest Asia	95.79%	15.53	8.88
Spain	97.57%	18.07	10.73
Spain Caucasoid	97.61%	18.11	10.79
Spain Other	6.30%	0.44	0.75
Sri Lanka	52.39%	4.16	1.47
Sri Lanka Asian	52.39%	4.16	1.47
Sudan	97.55%	17.43	10.38
Sudan Arab	70.21%	6.47	2.35
Sudan Black	2.19%	0.15	0.72
Sudan Mixed	97.79%	17.87	10.74
Sweden	100.00%	29.79	23.24
Sweden Caucasoid	100.00%	29.79	23.24
Taiwan	99.29%	20.37	14.21
Taiwan Oriental	99.29%	20.37	14.21
Thailand	96.83%	16.93	9.73
Thailand Oriental	96.83%	16.93	9.73
Tokelau	55.11%	4.24	1.56
Tokelau Polynesian	55.11%	4.24	1.56
Tonga	71.91%	6.16	2.49
Tonga Polynesian	71.91%	6.16	2.49
Tunisia	99.36%	20.76	14.54
Tunisia Arab	99.38%	20.85	14.6
Tunisia Berber	74.47%	6.6	2.74
Turkey	86.85%	10.04	5.32
Turkey Caucasoid	86.85%	10.04	5.32
Uganda	94.87%	13.76	8.3
Uganda Black	94.87%	13.76	8.3
Ukraine	50.64%	3.84	1.42
Ukraine Caucasoid	50.64%	3.84	1.42
United Arab Emirates	35.19%	2.54	1.08
United Arab Emirates Arab	35.19%	2.54	1.08
United States	99.89%	26.03	18.99
United States Amerindian	99.81%	19.31	14.13
United States Asian	99.46%	22.41	15.27
United States Austronesian	58.09%	4.8	1.67
United States Black	99.18%	21.74	14.67
United States Caucasoid	99.98%	29.12	22.47
United States Hispanic	99.71%	22.85	15.84
United States Mestizo	99.79%	22.98	16.05
United States Polynesian	99.88%	23.67	16.68
Venezuela	90.34%	9.48	7.05
Venezuela Amerindian	90.07%	9.17	7.01

Venezuela Caucasoid	11.45%	0.81	0.79
Venezuela Mestizo	9.75%	0.69	0.78
Venezuela Mixed	3.17%	0.22	0.72
Vietnam	96.27%	16.08	9.19
Vietnam Oriental	96.27%	16.08	9.19
Wales	1.00%	0.07	0.71
Wales Caucasoid	1.00%	0.07	0.71
West Africa	98.43%	19.67	12.42
West Indies	99.69%	23.55	16.2
Zambia	98.10%	14.99	9.71
Zambia Black	98.10%	14.99	9.71
Zimbabwe	98.03%	18.54	11.29
Zimbabwe Black	98.03%	18.54	11.29
**Average**	**82.86**	**14.22**	**9.35**
**Standard deviation**	**25.28**	**8.36**	**6.64**
