# Supplementary material for: A Comprehensive Computer Aided Vaccine Design Approach to Propose a Multi-Epitopes Subunit Vaccine against Genus Klebsiella Using Pan-Genomics, Reverse Vaccinology, and Biophysical Techniques
Source: Vaccines (Basel). 2021 Sep 27;9(10):1087. doi: 10.3390/vaccines9101087 (PMC8540426; doi:10.3390/vaccines9101087)
Supplement: Supplementary file 1 [file vaccines-09-01087-s001.zip › S-Table S3.pdf]

**TLR4** **Patchdock**

| Solution No | Score | Area | ACE | Transformation | PDB file of the complex |
|---|---|---|---|---|---|
| 1 | 17636 | 2196.4 | 411.82 | -2.73 1.41 -0.52 5.57 40.16 -36.28 | result.1.pdb |
| 2 | 17056 | 2719.9 | 435.53 | -1.74 0.96 0.85 -29.72 -24.55 -11.11 | result.2.pdb |
| 3 | 16948 | 2248.6 | 426.91 | -1.70 -0.24 -1.73 -13.03 24.85 19.06 | result.3.pdb |
| 4 | 16764 | 2566.6 | 401.38 | 1.22 -0.13 -1.80 18.23 23.27 5.55 | result.4.pdb |
| 5 | 16738 | 2254 | 420.99 | 0.77 0.59 3.08 55.35 7.69 -10.55 | result.5.pdb |
| 6 | 16400 | 2411.7 | 322.35 | 1.16 -0.01 -1.73 16.63 25.77 3.23 | result.6.pdb |
| 7 | 16154 | 2372.7 | 404.89 | -1.70 0.44 -2.89 44.20 32.76 30.39 | result.7.pdb |
| 8 | 16010 | 2056 | 382.30 | 2.44 -1.04 -1.55 63.19 10.64 5.20 | result.8.pdb |
| 9 | 15904 | 2199 | 153.84 | -0.24 0.99 2.11 24.66 8.70 -37.95 | result.9.pdb |
| 10 | 15732 | 1841.1 | 439.44 | 2.18 1.02 2.53 4.49 -0.59 -2.76 | result.10.pdb |
| 11 | 15724 | 2037.4 | 430.96 | -3.11 0.58 0.34 -20.17 20.72 10.91 | result.11.pdb |
| 12 | 15572 | 2365.7 | 463.65 | -1.94 0.82 -2.83 60.47 16.42 -17.14 | result.12.pdb |
| 13 | 15396 | 2543.1 | 350.02 | 1.57 -0.39 -1.25 -21.69 74.80 2.73 | result.13.pdb |
| 14 | 15372 | 2357 | 93.27 | 3.09 0.16 -1.23 -34.79 60.06 0.76 | result.14.pdb |
| 15 | 15370 | 2153.8 | 474.70 | -2.34 1.33 -1.94 33.31 29.30 -29.46 | result.15.pdb |
| 16 | 15290 | 2401.1 | 223.96 | 0.52 0.47 1.75 8.21 -13.23 -39.78 | result.16.pdb |
| 17 | 15170 | 2538.2 | 318.13 | 0.99 -0.87 2.66 50.86 -0.15 27.84 | result.17.pdb |
| 18 | 15168 | 1946.8 | 487.29 | -0.70 0.60 -1.42 22.41 49.31 -18.73 | result.18.pdb |
| 19 | 15144 | 2448.8 | 359.85 | 0.80 -0.36 1.40 -2.73 2.63 7.14 | result.19.pdb |
| 20 | 15112 | 2353.3 | 175.01 | 2.59 -0.07 2.82 -0.51 6.25 19.81 | result.20.pdb |
