# Supplementary material for: A Comprehensive Computer Aided Vaccine Design Approach to Propose a Multi-Epitopes Subunit Vaccine against Genus Klebsiella Using Pan-Genomics, Reverse Vaccinology, and Biophysical Techniques
Source: Vaccines (Basel). 2021 Sep 27;9(10):1087. doi: 10.3390/vaccines9101087 (PMC8540426; doi:10.3390/vaccines9101087)
Supplement: Supplementary file 1 [file vaccines-09-01087-s001.zip › S-Table S4.pdf]

MHC-I

Solution No	Score	Area	ACE	Transformation	PDB file of the complex
1	23626	3494.3	218.20	-0.21 0.73 1.85 21.24 -0.15 -38.99	result.1.pdb
2	18068	2967.1	188.91	-0.16 0.64 1.87 18.25 1.60 -39.95	result.2.pdb
3	17022	3179.6	358.75	1.79 -0.26 -1.04 -25.49 49.54 -25.39	result.3.pdb
4	16872	2745.3	232.04	2.43 0.03 -0.72 18.16 11.54 -13.99	result.4.pdb
5	16764	2410.1	-82.23	0.06 0.39 0.44 -18.44 -41.19 -37.56	result.5.pdb
6	16744	3259.5	378.20	-2.48 0.06 1.84 23.64 -11.33 -5.88	result.6.pdb
7	16534	2248.6	423.40	-1.01 0.90 -2.71 43.33 30.82 -39.70	result.7.pdb
8	16302	2436.8	399.72	-0.99 0.67 -2.88 55.08 24.40 -28.05	result.8.pdb
9	16250	2603.7	138.07	2.25 -0.32 -0.37 7.39 12.04 -15.56	result.9.pdb
10	16220	2520.2	-17.52	0.32 -0.16 0.73 -21.18 -44.83 -21.00	result.10.pdb
11	16198	2450.4	130.98	-1.95 1.06 -1.57 7.24 44.59 -43.49	result.11.pdb
12	16184	2497.9	141.54	0.14 0.87 1.61 12.56 3.77 -44.99	result.12.pdb
13	16166	3185.1	411.76	0.16 -0.61 0.20 -1.25 -3.72 -31.04	result.13.pdb
14	16148	2169.1	405.36	1.95 -0.53 -1.58 1.96 39.97 9.49	result.14.pdb
15	16008	2345.3	-72.45	0.62 -0.33 0.73 -26.25 -44.89 -18.87	result.15.pdb
16	15876	2435.1	498.39	2.75 -0.22 1.22 -29.11 -55.53 -32.27	result.16.pdb
17	15804	2113.1	489.02	1.82 -0.27 -2.97 26.82 -1.04 -34.50	result.17.pdb
18	15718	2314.5	180.05	-1.55 1.06 -2.06 22.59 40.58 -44.13	result.18.pdb
19	15608	2305.2	333.41	2.40 0.25 -1.02 -2.97 45.11 -52.97	result.19.pdb
20	15580	2174.1	15.03	2.09 0.10 -0.75 -27.78 35.56 -46.75	result.20.pdb
