# Supplementary material for: A Comprehensive Computer Aided Vaccine Design Approach to Propose a Multi-Epitopes Subunit Vaccine against Genus Klebsiella Using Pan-Genomics, Reverse Vaccinology, and Biophysical Techniques
Source: Vaccines (Basel). 2021 Sep 27;9(10):1087. doi: 10.3390/vaccines9101087 (PMC8540426; doi:10.3390/vaccines9101087)
Supplement: Supplementary file 1 [file vaccines-09-01087-s001.zip › S-Table S5.pdf]

MHC-II

Solution No	Score	Area	ACE	Transformation	PDB file of the complex
1	17500	2213.1	92.40	0.93 -0.60 0.53 -51.27 -40.87 -4.29	result.1.pdb
2	17352	2490.7	-163.68	0.36 0.12 -1.13 11.77 -4.65 -11.55	result.2.pdb
3	17290	2791	-297.23	0.22 0.28 -1.23 9.19 -10.50 -12.28	result.3.pdb
4	17236	2287.9	407.96	0.34 0.49 -1.32 -17.86 31.95 -26.21	result.4.pdb
5	16498	2338.4	-22.50	-1.00 -0.27 2.49 0.38 -5.34 -9.78	result.5.pdb
6	16122	2393.3	187.16	-0.58 0.55 3.12 27.45 19.01 -22.11	result.6.pdb
7	15900	2108.1	105.34	0.17 1.41 -1.58 -47.37 -1.78 -47.67	result.7.pdb
8	15856	1997.4	196.65	-0.83 -0.59 0.34 -16.35 -10.85 52.61	result.8.pdb
9	15734	1973.9	-10.49	-1.79 0.40 -2.26 -21.31 18.18 -13.51	result.9.pdb
10	15682	2702.1	8.48	-3.02 0.33 1.09 6.00 -12.13 17.26	result.10.pdb
11	15646	2500.4	70.11	-0.15 0.66 1.98 19.18 -27.07 -29.93	result.11.pdb
12	15628	2294.7	113.58	1.26 -0.77 0.75 -17.44 -33.66 14.68	result.12.pdb
13	15626	2864.6	107.42	-1.75 -1.10 2.73 2.50 1.57 -3.23	result.13.pdb
14	15550	2253.8	430.24	0.41 0.28 -1.24 -20.24 5.54 -37.06	result.14.pdb
15	15382	2393.2	-94.82	2.76 0.15 0.94 0.06 -6.12 14.51	result.15.pdb
16	15112	2036.4	11.61	-0.90 -0.87 3.12 16.01 -1.70 23.94	result.16.pdb
17	15044	1831.4	204.53	1.11 -0.63 0.78 -46.08 -46.93 -2.79	result.17.pdb
18	15012	2205.4	-161.73	-1.87 0.66 -2.35 -18.17 15.50 -11.90	result.18.pdb
19	14944	2233.7	-66.64	3.10 -0.16 -1.71 9.70 44.03 -3.16	result.19.pdb
20	14904	2448.2	198.30	-0.25 -0.24 -0.98 -34.50 4.37 -20.47	result.20.pdb
